# Bio‐Inspired Micropatterned Platforms Recapitulate 3D Physiological Morphologies of Bone and Dentinal Cells

**DOI:** 10.1002/advs.201801037

**Published:** 2018-10-12

**Authors:** Chi Ma, Bei Chang, Yan Jing, Harry Kim, Xiaohua Liu

**Affiliations:** ^1^ Department of Biomedical Sciences Texas A&M University College of Dentistry Dallas TX 75246 USA; ^2^ Department of Orthodontics Texas A&M University College of Dentistry Dallas TX 75246 USA; ^3^ Department of Orthopaedics Texas Scottish Rite Hospital for Children University of Texas Southwestern Medical Center TX 75219 USA

**Keywords:** 3D, cell morphology, micropatterns, odontoblasts, osteocytes

## Abstract

Cells exhibit distinct 3D morphologies in vivo, and recapitulation of physiological cell morphologies in vitro is pivotal not only to elucidate many fundamental biological questions, but also to develop new approaches for tissue regeneration and drug screening. However, conventional cell culture methods in either a 2D petri dish or a 3D scaffold often lead to the loss of the physiological morphologies for many cells, such as bone cells (osteocytes) and dentinal cells (odontoblasts). Herein, a unique approach in developing a 3D extracellular matrix (ECM)‐like micropatterned synthetic matrix as a physiologically relevant 3D platform is reported to recapitulate the morphologies of osteocytes and odontoblasts in vitro. The bio‐inspired micropatterned matrix precisely mimics the hierarchic 3D nanofibrous tubular/canaliculi architecture as well as the compositions of the ECM of mineralized tissues, and is capable of controlling one single cell in a microisland of the matrix. Using this bio‐inspired 3D platform, individual bone and dental stem cells are successfully manipulated to recapitulate the physiological morphologies of osteocytes and odontoblasts in vitro, respectively. This work provides an excellent platform for an in‐depth understanding of cell–matrix interactions in 3D environments, paving the way for designing next‐generation biomaterials for tissue regeneration.

Cells in the body reside in a distinct 3D microenvironment – highly structured extracellular matrix (ECM) that is a nanofibrous network and possesses well‐organized hierarchic architecture ranged from nano to macro scales. When cells are removed from the in vivo microenvironment and are cultured on an artificial matrix in vitro, the cells usually cannot retain their 3D physiological morphologies. For instance, osteocytes are star‐shaped bone cells with dendritic processes extending from a small chamber called a lacuna to many minute channels called canaliculi.^1^ When the osteocytes were extracted from a bone tissue and cultured on a 2D tissue culture plastic, the cells rapidly lost the characteristic dendritic shapes, leading to the changes of gene expressions.^2^, ^3^ Similarly, an odontoblast in the pulp chamber of a tooth has a long process that extends deeply into a dentinal tubule, and the odontoblast loses its long process when cultured on a petri dish.^4^, ^5^

Since cell morphology (or cell shape) is one of the crucial factors to regulate many biological processes, including stem cell commitment and selective differentiation,^6^, ^7^ recapitulation of the physiological cell morphology in vitro is essential to elucidate these fundamental biological questions, leading to the development of new approaches for tissue regeneration and drug screening. To do that, a bio‐inspired 3D platform that is capable of precisely mimicking both the hierarchic architecture and the compositions of the ECM needs to be developed to accommodate the cells extracted from a tissue or organ.

Bone and dentin are mineralized tissues and their ECMs have a hierarchic structure in which the well‐defined tubules/canaliculi, which have a diameter ranged from several hundred nanometers to a few micrometers, are embedded in a highly interconnected nanofibrous 3D network.^1^, ^8^ Reconstructing such a hierarchic architecture using biodegradable materials and integrating it into a biomimetic 3D platform is a considerable challenge. Conventional approaches using synthetic hydrogels and collagen‐based matrix can form 3D fibrous network,^9^, ^10^, ^11^ however; those scaffolding materials are not capable of mimicking the tubular microstructures of the ECMs of bone and dentin tissues. Consequently, to date, there are no suitable platforms that are capable of recapitulating the morphologies of osteocytes and odontoblasts in vitro.

Micropatterning approaches, which include photolithography, ink‐jet printing, microcontact printing, soft lithography, and self‐assembly, are widely used to control cell–material interactions within a microdomain,^12^, ^13^, ^14^, ^15^, ^16^ and is a potential tool to fabricate bio‐inspired platforms. Currently, most of the micropatterning methods are limited to fabricate 2D substrates using non‐biodegradable materials, such as polydimethylsiloxane, polyacrylamide, and polystyrene, and cannot mimic the 3D microstructure of the ECM.^17^, ^18^, ^19^ More importantly, none of the micropatterned substrates can truly recapitulate the components and the hierarchic architecture of the ECMs of bone or dentin.

Herein, we report a unique approach to developing a 3D ECM‐like micropatterned matrix as a physiologically relevant 3D platform to recapitulate the bone and dentin cell morphologies in vitro. The bio‐inspired micropatterned matrix precisely mimics the hierarchic 3D tubular/canaliculi architecture as well as the compositions of the ECM of mineralized tissues. In addition, the synthetic micropatterned matrix is capable of precisely controlling one single cell in a microisland, providing an excellent platform to study cell–matrix interactions. Using this bio‐inspired 3D platform, for the first time, we successfully manipulated individual bone and dental stem cells to recapitulate the physiological morphologies of osteocytes and odontoblasts, respectively.

**Scheme** **1** illustrates the steps for the preparation of the bio‐inspired 3D platform. It started from the fabrication of a nanofibrous matrix using an electrospinning process, which is a remarkably simple, robust, and versatile technique to generate ECM‐like nanofibrous structure (Scheme 1A). We selected methacrylate‐modified gelatin (GelMA) as the substrate biomaterials for three reasons. First, gelatin is derived from collagen (the main component of natural ECM) by hydrolysis; therefore, is an ideal biomaterial to mimic the composition of natural ECM.^20^, ^21^ Because gelatin is a denatured protein, the denaturing hydrolysis process eliminates the potential risk of pathogens transmission associated with collagen, making it an excellent biomaterial for tissue engineering. Second, gelatin is transparent in aqueous solution and allows us to conveniently monitor cells that are embedded in the gelatin matrix using optical microscopies, including confocal laser scanning microscopy.^22^, ^23^ Third, GelMA is chemically modified gelatin with double bonds, which facilitates chemical crosslinking with polyethylene (glycol) diacrylate (PEGDA) to create a stable cellular non‐adhesive background. After the electrospining process, the GelMA nanofibrous matrix was crosslinked with carbodiimide in a solvent mixture (acetone/water = 9*5*/05 v/v) to preserve the nanofibrous structure (Scheme 1B). In the next step, PEGDA was cast onto the surface of the crosslinked GelMA matrix (Scheme 1C), followed by a UV‐induced photolithography process to create a micropatterned matrix (Scheme 1D). The alkene groups on the PEGDA and GelMA were initiated and crosslinked together to stabilize the micropattern. Finally, a computer‐aided laser ablation technology was carried out using a Leica LMD 7000 system to create a 3D nanofibrous micropatterned tubular matrix (Scheme 1E).

**Scheme 1:**
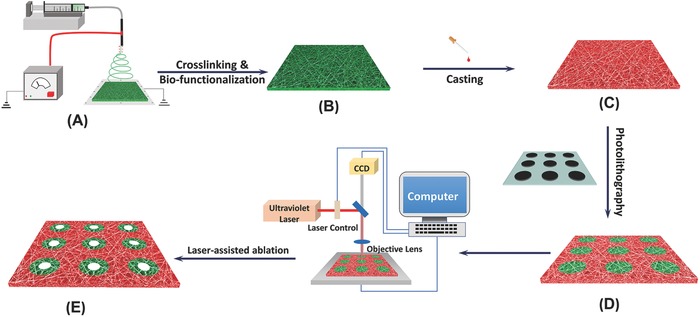
Illustration of the fabrication of a nanofibrous micropatterned tubular 3D platform.

As shown in **Figure** **1**A–C, nanofibrous micropatterned gelatin microislands were successfully created by combining the electrospinning and photolithography processes. The microislands were composed of gelatin nanofibers with an average diameter of approximately 200 nm, which is at the same range of collagen fibers in natural ECM. The shape and size of microislands were precisely controlled by the photomasks, and Figure 1A shows well‐organized circular microislands with a diameter of 60 µm. The PEGDA hydrogel interpenetrated with the nanofibrous gelatin matrix and covered the substrate surfaces that surrounded the microislands. A unique laser ablation process was performed to form 3D tubular microislands, which mimic the hierarchical structures of natural dentin and bone matrix. Specifically, Figure 1D,E is the synthetic tubular matrix with one tubule in a microisland, which had the same architecture and similar composition of natural dentin matrix (Figure 1F). Furthermore, the size of the tubule in the synthetic tubular matrix was approximate 3 µm, which is the same as that of a natural dentin tubule. Figure 1G,H is the synthetic tubular matrix with multiple tubules in a microisland, which resembled the architecture and composition of natural bone matrix (Figure 1I).

**Figure 1:**
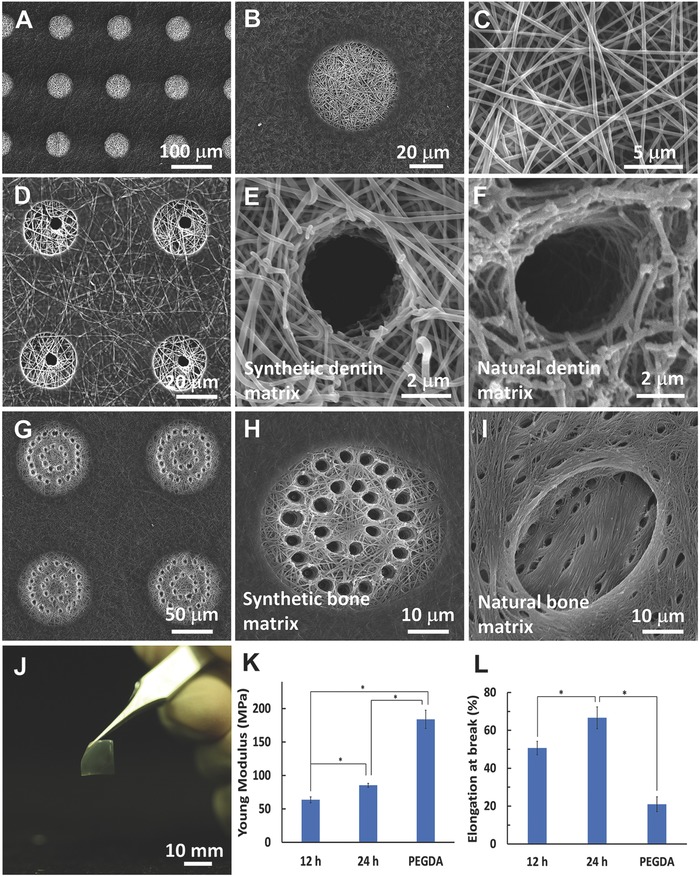
A) SEM image of a nanofibrous micropatterned gelatin matrix with circular microislands of 60 µm in diameter. B) The magnified image of (A), showing the nanofibrous microisland surrounded with cell‐repellent PEG. C) The enlarged image of (B), showing gelatin nanofibers in the microisland. D) SEM image of a 3D nanofibrous micropatterned matrix with one tubule in each microisland. E) The magnified image of (D), showing the microisland has a 3D nanofibrous tubular architecture, which precisely mimics the natural tubular structure of F) dentinal ECM. G) SEM image of a 3D nanofibrous micropatterned matrix with multiple tubules in each microisland. H) The magnified image of (G), showing the microisland has a 3D nanofibrous tubular architecture, which aims at resembling the natural structure of I) bone ECM. J) A photographical image of a micropatterned gelatin matrix, showing the matrix is transparent and easy to handle. K) The Young's moduli of the nanofibrous gelatin matrices with different crosslinking times (12 and 24 h) and with the addition of PEGDA onto the micropatterned gelatin matrix. L) The elongation at break of the gelatin nanofibrous matrix with different crosslinking time (12 and 24 h) and with the addition of PEGDA onto the micropatterned matrix (*n* = 5, *p < 0.05).

We developed this innovative laser‐guided ablation approach, which is a noncontact, high‐precision, and computer programming of machining process, to introduce 3D microstructure into nanofibrous matrices. Using this approach, the tubular size, distribution, and density were precisely controlled to match those of natural dentin and bone ECMs. The laser power, laser writing speed, and pulse frequency were the major factors to control the tubular structure. Specifically, the laser power was used to control the size and the depth of the tubular pores, and the laser writing speed and laser pulse frequency were used to adjust the distance between the tubules. Small tubules (<10 µm) were regenerated with low laser power (<20 µJ), and narrow spaces between the tubules were obtained from high pulse frequencies and low writing speed (Figure S1, Supporting Information). However, when a high laser frequency or a low writing speed was used, the tubules would be interconnected and form microgrooves (Figure S1D, Supporting Information, where the writing speed = 1200 µm s^−1^ and the frequency = 40 Hz). Because the laser ablation approach was precisely modulated by a computer programing, the depth of the tubules could be readily controlled by the repetition of the laser ablation process. In addition, the orientation of the tubules to the matrix was conveniently adjusted via the angle between the laser and the matrix plane (Figure S2, Supporting Information). Within the range of the power scale in our experiments, the surface chemistry of the tubular matrix mostly remained intact, as indicated by the element compositions of carbon, nitrogen, and oxygen in the matrix (Figure S3, Supporting Information).

The 3D tubular gelatin matrix had excellent mechanical properties (Figure 1J). Both the Young's modulus and the elongation at break of the micropatterned matrix increased with the crosslinking time (Figure 1K,L). More strikingly, the incorporation of PEGDA with the gelatin matrix increased the mechanical strength from 88 ± 5 to 184 ± 27 MPa, which was due to the crosslinking of the PEGDA with GelMA. While the crosslinking of the PEGDA with GelMA reduced the elasticity of the matrix, the elongation at break was still more than 21%, which was appropriate to be used as a cell culture substrate.

Since the 3D tubular gelatin matrix had almost the same compositions to those of collagen (the major organic component of the ECM in bone and tooth tissues), the mechanical property of the gelatin matrix was similar to that of the decalcified bone/tooth tissues. To further increase the mechanical strength of the tubular gelatin matrix, a simulated body fluid incubation process, which was developed in our previous study,^20^ can be adopted to incorporate bone‐like apatite onto the surface of the biomimetic 3D tubular gelatin matrix.

We chose human dental pulp stem cells (DPSCs) as a model cell type and examined how the DPSCs interacted with the nanofibrous micropatterned matrix. The DPSCs quickly attached to the microislands after they were seeded onto the micropatterned matrix. Within 1 h, the cell started to spread in the microisland, and reached to a stable stage 24 h after cell seeding (Movie S1, Supporting Information and Figure S4, Supporting Information). Regardless of the size of the microisland, the micropatterned matrix strictly confined the DPSCs within the nanofibrous microislands, confirming the strong cell‐repellent effect of the PEG on the micropatterned matrices (**Figure** **2**A,C). While the DPSC in a smaller microisland was less spreading, it had a higher cell height on the microisland (Figure 2D).

**Figure 2:**
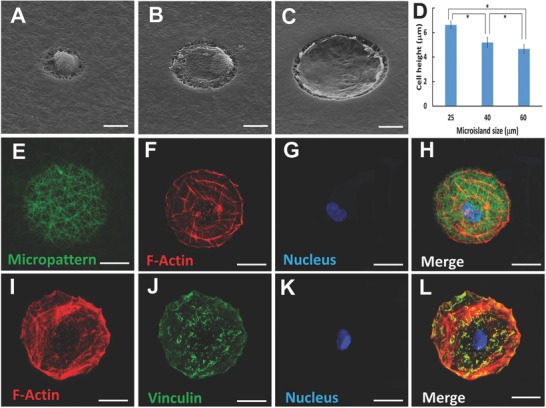
A–C) Typical morphologies of human DPSCs on the microislands with different sizes. A) 25, B) 40, and C) 60 µm. D) The average cell height on the microislands with different sizes. The cell heights were calculated from the z stack of the confoal images (*n* = 50, *p < 0.05). E–H) Confocal images of a DPSC cultured on the FITC‐labeled microisland (60 µm in diameter). E) Gelatin nanofibers in the microisland. F) F‐actin of the DPSC. G) The nucleus of the DPSC. H) The confocal image that merges the nanofibers, F‐actin and nucleus. I–K) Focal adhesion images of a DPSC adhesion on a microisland (60 µm in diameter). I) F‐actin of the DPSC, showing the cytoskeleton of the DPSC. J) The distribution of vinculins of the DPSC on the microisland. K) The nucleus of the DPSC. L) The confocal image that merges the F‐actin, vinculin, and nucleus.

Fluorescent images were used to further examine DPSC adhesion on the bio‐inspired microislands. To make the nanofibrous microisland visible under fluorescence microscopy, the fluorescein isothiocyanate (FITC) labeled gelatin was added during preparation of the nanofibrous matrix. Since the UV light quenched the FITC molecules surrounding the microislands during the process of the photolithography, the fluorescent microislands were obtained (Figure 2E). F‐actin, which was stained with red color to present the cytoskeleton, clearly showed the widespread morphology of the DPCS within the microisland (Figure 2F), consistent with the SEM observation (Figure 2A–C).Vinculin, a key protein of focal adhesion complex, is an indicator to evaluate the formation of focal adhesion. As shown in Figure 2–L, the expression of vinculin was detected both at the edge and in the middle of the microisland, revealing the strong interaction between the DPSC and the nanofibers.

The microislands with different sizes had considerably high cell occupation ratios (**Figure** **3**A–F). Furthermore, the cell occupation ratio on the microislands increased with the microisland size. In addition, the addition of seeding times increased cell numbers on the microislands (Figure 3G). For example, more than 70.7% of the microislands with the size of 60 µm were occupied by the DPSCs when they were seeded twice.

**Figure 3:**
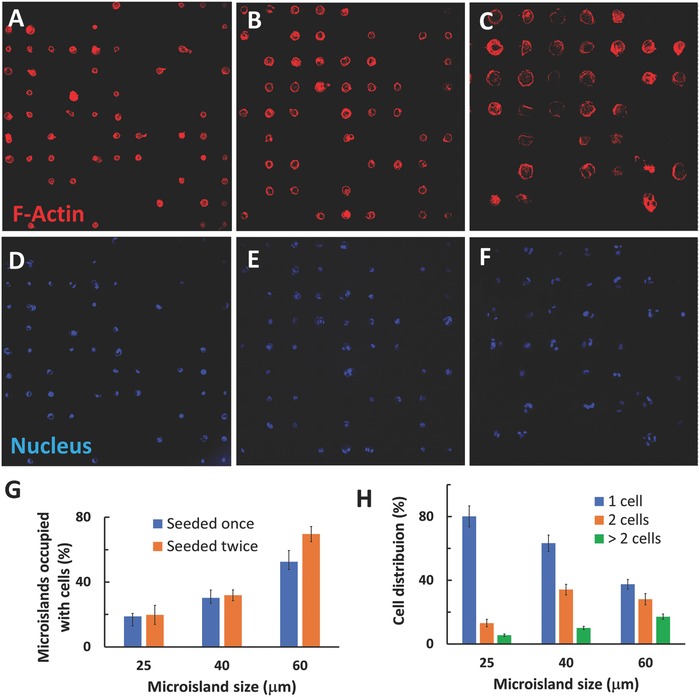
Confocal images of DPSCs after cultured on the microislands of different sizes for 1 week. The DPSCs were stained with phalloidin (red) to show the cells occupying the circular microislands with the diameters of A) 25, B) 40, and C) 60 µm. D–F) The DPSCs were stained with DAPI to show single or multiple cells in one microisland with the diameter of D) 25, E) 40, and F) 60 µm. G) The effects of microisland diameters and seeding times on the ratios of micropatterns occupies with cells. H) The cell number distribution in each microisland with different diameters. For (G) and (H), five region of interests (ROIs) and at least 100 microislands in each ROI were selected in each group for the analyses.

The cell numbers on each microisland were controlled by the size of microislands. For instance, among the microislands resided by DPSCs, over 80.2% of the microislands with the size of 25 µm was occupied by single cells (Figure 3H). In contrast, only 37.5% of the microislands with the size of 60 µm were occupied by single cells. Accordingly, the ratios of two cells and more than two cells in each microisland increased with the size of the microisland. The micropatterned matrices had excellent capability to constrain stem cells within the microislands. Moreover, even the cells were restricted to proliferate, most of the cells in the microislands stayed alive after they were cultured for 4 weeks, confirming the high biocompatibility of the micropatterned gelatin matrix (Figure S5, Supporting Information), which was difficult to achieve when using other micropatterned surfaces.^24^

One of the advantages of the biomimetic micropatterned gelatin matrix was the flexibility of being functionalized with bioactive molecules. Besides having cell adhesion motif, the GelMA possesses free amino groups, which allows the GelMA microislands to readily conjugate with proteins or peptides using a simple carbodiimide crosslinking chemistry. We selected bone morphogenetic protein 2 (BMP‐2) as an example, and grafted BMP‐2 onto the nanofibers of the microislands using a one‐step process with a heterobifunctional crosslinker that contains both *N*‐hydroxysuccinimide ester and maleimide groups (Scheme S1, Supporting Information). To detect the distribution of the coupled BMP‐2 on the microislands, the BMP‐2 antibody was added for immunofluorescent staining. It was shown in **Figure** **4**A–D that the BMP‐2 was strictly confined in the microislands. The BMP‐2 was evenly distributed in the microislands with a density of 80 ng cm^−2^, which could be easily modulated by the reactant concentration during the crosslinking process. The cell adhesion ratio on the microislands was enhanced after the conjugation of BMP‐2 onto microislands (Figure S6, Supporting Information). Alkaline phosphatase (ALP) is an odontogenic differentiation marker of DPSCs, and the ALP assay was performed to evaluate the bioactivity of the BMP‐2 incorporated in the microislands. As shown in Figure 4E–G, the DPSC in the microisland exhibited a much stronger ALP expression than the control group, indicating the high bioactivity of the BMP‐2 conjugated onto the microislands. It should be noted that other proteins and peptides can also be incorporated onto the nanofibrous micropatterned matrix using the same conjugating approach.

**Figure 4:**
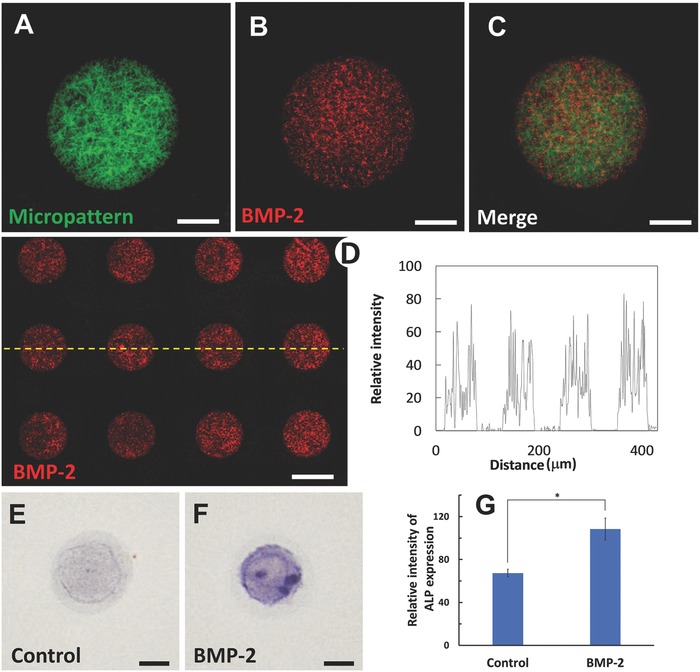
Conjugation of BMP‐2 onto the nanofibers of the microislands. A) FITC‐labeled nanofibers on the microisland of a diameter of 60 µm. B) The BMP‐2 (red) on the microisland. C) The merged image of (A) and (B). D) The distribution of BMP‐2 on the micropatterned gelatin matrix. The values of the relative intensity indicate that the BMP‐2 can only be detected inside the microislands. E) The typical ALP staining image of a DPSCs cultured on a microisland in an odontogenic differentiation medium for 3 d. F) The typical ALP staining image of a DPSC cultured on a BMP‐2 coupled microisland in an odontogenic differentiation medium for 3 d. G) The relative intensity of the ALP expression of the DPSCs cultured on the microislands with/without BMP‐2 conjugation (*n* = 10, *p < 0.05). Scale bar: 20 µm.

The micropatterned gelatin matrix was transparent and could be further combined with cytological section techniques to obtain high‐quality images at the lateral view of cells on the microislands, which was very difficult, if not impossible, for the micropatterns prepared from other materials, such as glass, silicon, or polystyrene. **Figure** **5**A–C showed that all the cell bodies of the DPSCs remained on the surface of the microislands, while the short pseudopodia inserted into the nanofibrous matrix. Overall, the nanofibrous microislands were a 2D matrix and did not allow the DPSC to exhibit in vivo like cell morphology.

**Figure 5:**
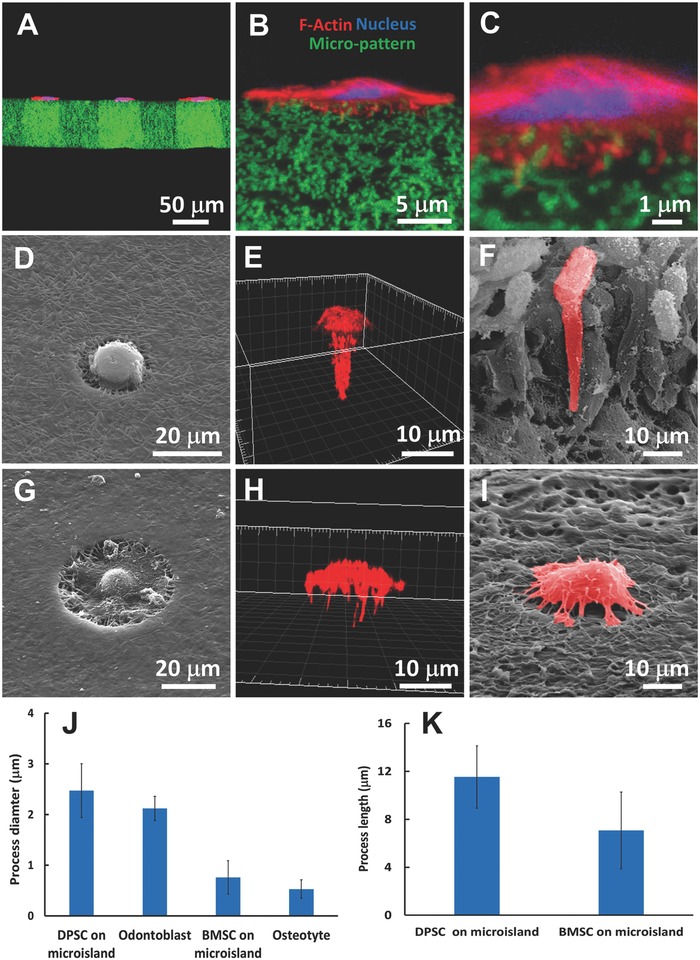
A) The cross‐section overview of DPSCs cultured on a micropatterned matrix. B) The cross‐section view of a DPSC cultured on a microisland. C) The enlarged confocal image of B), showing the short pseudopodia of the DPSC inserted into the nanofibrous matrix. D) The typical SEM image of a DPSC cultured on a 3D microisland with a single tubule. E) The 3D reconstructed morphology of the DPSC on the 3D microisland. F) The morphology of odontoblasts in vivo. G) The typical SEM image of a BMSC cultured on a 3D microisland with multiple tubules. H) The 3D reconstructed morphology of the BMSC on the 3D microisland. I) The morphology of an osteocyte in vivo. J) The cellular process diameters of DPSC and BMSC on the 3D microislands, and the cellular process diameters of human odontoblast and mouse osteocyte. K) The cellular process lengths of DPSC and BMSC on the 3D microislands.

To stimulate the formation of in vivo like morphologies of odontoblasts and osteoblasts, 3D nanofibrous tubular microislands were prepared and seeded with DPSCs and BMSCs. Specifically, the DPSC was seeded on a microisland that had a single tubule with a diameter of 3.0 µm, and the BMSC was seeded on a microisland that had 28 tubules with an average diameter of 1.8 µm. After being cultured on the biomimetic 3D microislands for 48 h, the morphologies of the DPSC and BMSC were showed in Figure 5D,I. The DPSC was highly polarized with a long process inserting into the tubule, while the cell body displayed spherical shape aligning on the surface of the microisland (Figure 5D,E), which resembled the 3D morphology of the odontoblast in the body (Figure 5F). Similarly, the cell body of the BMSC attached to the surface of tubular microisland, while a number of dendrite‐like structures were inserted into the tubules of the microisland (Figure 5G,H). Overall, the BMSC on the microisland had the same 3D morphology of the osteocyte in the body (Figure 5I). Semiquantitative analyses further showed that the DPSC on the tubular microisland had the cellular process with a diameter of 2.4 µm, similar to the process of human odontoblast in vivo (2.2 µm, Figure 5J). Also, the BMSC on the multicanaliculi microisland had the cellular dendrites with an average diameter of 0.7 µm, similar to the dendrites of the human osteocyte (0.5 µm, Figure 5J). In addition, the average process lengths of the DPSC and BMSC on the microislands were 11.5 and 7.1 µm, respectively (Figure 5K). It is expected that the process lengths of the DPSC and BMSC would further increase with extended culturing time and longer tubules in the micropatterned matrix. Those results clearly show the pivotal role of the biomimetic 3D platform in guiding the formation of physiological morphologies of osteocytes and odontoblasts. It should be noted that the bio‐inspired micropatterned 3D matrix is also an excellent platform to recapitulate the 3D physiological morphologies of many other types of cells, such as neuron and dendritic cells.

In summary, we developed a unique approach to build bio‐inspired 3D micropatterned matrices that precisely mimic the hierarchic architecture and compositions of natural ECM. Using the ECM‐like micropatterned tubular matrices as a template, we, for the first time, recapitulated the physiological morphologies of bone and dentinal cells in vitro. The micropatterned matrix is an excellent platform for an in‐depth understanding of cell–matrix interactions in 3D environment, which will not only help elucidate many fundamental biological processes, but also guide the design of next‐generation biomaterials for tissue repair and regeneration.

## Experimental Section

*Fabrication of Nanofibrous Gelatin Matrix*: GelMA and FITC‐labeled gelatin were synthesized as previously described.^25^, ^26^ An electrospinning process (Spraybase platform, Ireland) was carried out to prepare nanofibrous gelatin matrix at room temperature. The electrospinning solution was prepared by dissolving the GelMA into the mixed solvents of hexafluoroisopropanol/acetic acid/ethyl acetate/water (5/2.5/1.5/1) with a concentration of 20% (w/v). A voltage of 12 kV and a feeding rate of 0.5 mL h^−1^ were used to fabricate the gelatin nanofibers, which were collected onto a drum of 30 cm in diameter with a rotation rate of 80 rpm. The distance between the drum collector and the spray tip was 10 cm. After collecting a thickness of approximately 100 µm, the nanofibrous gelatin matrix was crosslinked with carbodiimide in a solvent mixture (acetone/water = 95/5 (v/v)). To visualize nanofibrous matrix under fluorescent microscopy, the FITC‐labeled gelatin (2%) was added into the GelMA solution.

*Fabrication of Patterned Microislands*: The gelatin matrix was cut to a size of 1 cm × 1 cm and was mounted on a glass slide. Ten microliters of PEGDA aqueous solution (20%) including 1% of 2‐hydroxy‐4′‐(2‐hydroxyethoxy)‐2‐methylpropiophenone) was added to the gelatin matrix. Next, a photomask (Digidat, Inc. CA, USA) was covered on the gelatin matrix, and was exposed to a UV light with a power of 15 mW cm^−2^ for 1 min. The micropatterned matrix was incubated in distilled water for 1 h to remove the unreacted PEGDA, and was dehydrated in ethanol, and vacuum dried for later use.

*Conjugation of BMP‐2 onto the Nanofibers of the Microislands*: 4‐(*N*‐Maleimidomethyl) cyclohexane‐1‐carboxylic acid 3‐sulfo‐*N*‐hydroxysuccinimide ester sodium salt (Suflo‐SMCC) was used to conjugate BMP‐2 onto the gelatin nanofibers. After 4 mg of Suflo‐SMCC was dissolved in 1 mL of phosphate‐buffered saline (PBS), the gelatin matrix was added into the Suflo‐SMCC solution for 1 h at room temperature. After the activation process, the gelatin matrix was washed with PBS for three times, and incubated in 100 mL of BMP‐2 solution (50 µg mL^−1^) at 4 °C for 1 h. The resulting nanofibrous matrix was washed with PBS and air‐dried.

*Fabrication of 3D Nanofibrous Micropatterned Tubular in Microislands*: To generate 3D tubular microislands, the micropatterned matrix was paved and dried on a glass slide (Figure S7A, Supporting Information). The microislands were visible under a LMD 7000 system (Figure S7B, Supporting Information). The array of the tubules was programmed with the software of the LMD 700 (Leica micro‐dissection V7.5.1). The tubules within the microislands were generated via a laser ablation process, and the sizes of the tubules were controlled by the laser power and laser frequency (Figure S7C, Supporting Information).

*Cell Experiments*: Human dental pulp stem cells (DPSCs) were a gift from Dr. Songtao Shi, The University of Pennsylvania School of Dentistry. The cells were isolated from surgical waste (extracted human wisdom teeth) that was approved by IRB (Protocol# USC IRB #HS‐07‐00701). Informed signed consent was obtained from the volunteer. Human bone marrow stem cells (BMSCs) were purchased from Lonza. Both the DPSCs and BMSCs were cultured in an ascorbic acid‐free α‐modified essential medium (a‐MEM; GIBCO, Invitrogen, Carlsbad, CA) supplemented with 10% fetal bovine serum (FBS; Invitrogen) and 1% penicillin–streptomycin (Invitrogen) in a humidified incubator with 5% CO_2_ at 37 °C. The culture medium was changed every 2 days, and the DPSCs and BMSCs of passages 3–5 were used for the experiments. To test cell selectivity on microislands, 2 × 10^4^ of cells were seeded onto 1 cm^2^ of microislands. One hour after cell seeding, the unattached cells were washed off the matrix by gently pipetting up the culture medium. When necessary, a secondary seeding process was conducted 1 h after the first seeding step. An odontogenic medium was used to test the ALP activity of the DPSCs on the microislands.^27^

For F‐actin staining, the cell‐microisland construct was rinsed with Dulbecco's phosphate‐buffered saline (DPBS) and fixed with 4% paraformaldehyde in PBS for 30 min. The staining process was operated following with the manufacturer's instruction (CF633 phalloidin, Biotium, 00046), then followed by the staining of nuclei with Hoechst 33342 (1 µg mL^−1^) for 15 min and washed with PBS for three times.

For immunofluorescence staining of vinculin, the samples were blocked in 5% goat serum (Gibco) for 4 h at room temperature, and were reacted with antivinculin antibody (1:150, Abcam ab129002) and CF633 phalloidin (10 U mL^−1^, Biotium, 00046) overnight. After being washed with PBS for three times, the samples were stained with Alexa Fluor Plus 555 secondary antibody (1:200, Invitrogen, A32732) for 2 h, followed by 1 µg mL^−1^ Hoechst 33342 for 20 min. The samples were washed and mounted with coverslip (CoverWell Imaging Chamber Gasket).

For confocal observation, directly mounted samples were used for taking top‐view images. Three samples including at least 300 microislands were collected to measure the cell occupation ratio (the percentage number of microislands that are occupied by cells) and cellular spreading area within each microisland. To obtain high‐quality images of the lateral view of cells, the stained samples were embedded and processed with freezing microtome section. The sections with a thickness of 30 µm were harvested and immediately mounted with coverslips for confocal observations. At least 30 lateral images from three samples were collected and measured for the lengths and diameters (the diameters at the half lengths) of the cellular processes. High‐resolution images were taken using the stack mode with each step of 400 nm. The image files were exported for 3D reconstruction in Imaris 9.0.

ALP staining was operated following the manufacturer's instruction. Briefly, the samples were washed with PBS for 2 min, and fixed with 60% citrate buffered acetone for 5 min. The samples were immersed into an ALP staining solution for 30 min. After that, the samples were washed and stained with Hoechst 33342 for 20 min. The relative activity of the ALP was measured and analyzed following a previous report.^17^

For SEM observation, each sample was dehydrated with graded ethanol solutions (50%, 70%, 95%, 100%, 30 min for each) and dried in a vacuum oven. The dried samples were coated with gold using a sputter coater (SPI‐module Sputter Coater Unit, SPI Supplies/Structure Probe, Inc.) and observed under a SEM machine (JSM6010, JEOL).

*Statistical Analysis*: Quantitative results were presented as mean ± standard deviation. The unpaired Student's *t*‐test was used to test the significance between two groups, and the analysis of variance test was applied for multiple group comparisons. A value of *P* < 0.05 was considered statistically significant.

## Conflict of Interest

The authors declare no conflict of interest.

## Supporting information

Supplementary

Supplementary
