# Supplementary material for: Bio‐Inspired Micropatterned Platforms Recapitulate 3D Physiological Morphologies of Bone and Dentinal Cells
Source: Adv Sci (Weinh). 2018 Oct 12;5(12):1801037. doi: 10.1002/advs.201801037 (PMC6299721; doi:10.1002/advs.201801037)
Supplement: Supplementary file 1 — Supplementary [file ADVS-5-1801037-s002.pdf]

Copyright WILEY-VCH Verlag GmbH & Co. KGaA, 69469 Weinheim, Germany, 2018.

ADVANCED SCIENCE
Open Access

Supporting Information

for *Adv. Sci.,* DOI: 10.1002/advs.201801037

Bio-Inspired Micropatterned Platforms Recapitulate 3D Physiological Morphologies of Bone and Dentinal Cells

*Chi Ma, Bei Chang, Yan Jing, Harry Kim, and Xiaohua Liu**

Copyright WILEY-VCH Verlag GmbH & Co. KGaA, 69469 Weinheim, Germany, 2016.

Supporting Information

Bio-inspired Micropatterned Platforms Recapitulate 3D Physiological Morphologies of Bone and Dentinal Cells

*Chi Ma, Bei Chang, Yan Jing, Harry Kim, and Xiaohua Liu**

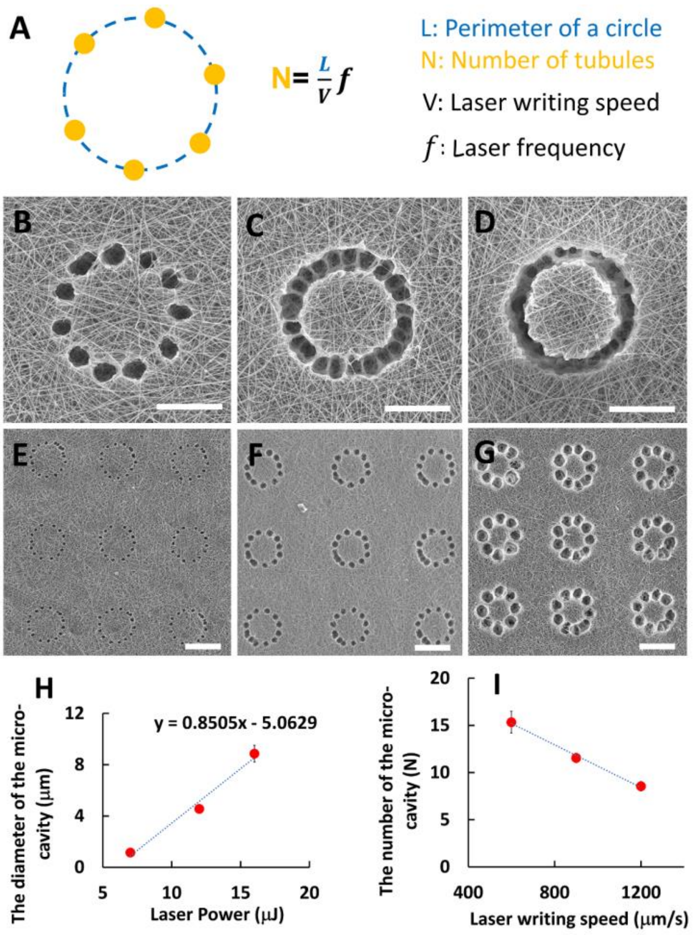

Figure S1. **(A)** Illustration of fabricating 3D tubular gelatin matrix with different laser writing speeds and laser frequencies. (**B-D**) SEM images of the 3D microstructures with the laser power = 12 μJ, the laser frequency = 40Hz, and the laser writing speed of **(B)** 600 μm/s, **(C)** 900 μm/s and **(D)** 1200 μm/s. (**E-G**) SEM images of the 3D microstructures with the laser writing speed = 900 μm/s and different laser frequencies and laser power. **(E)** Laser frequency = 60 Hz, laser power = 7 μJ; **(F)** Laser frequency = 40 Hz, laser power = 12 μJ; **(G)** Laser frequency = 20Hz, laser power = 16 μJ. **(H)** The relationship between the laser power and the micro-cavity diameters; **(I)** The relationship between the micro-cavity numbers and the laser writing speeds. Scale bars: 20 μm.

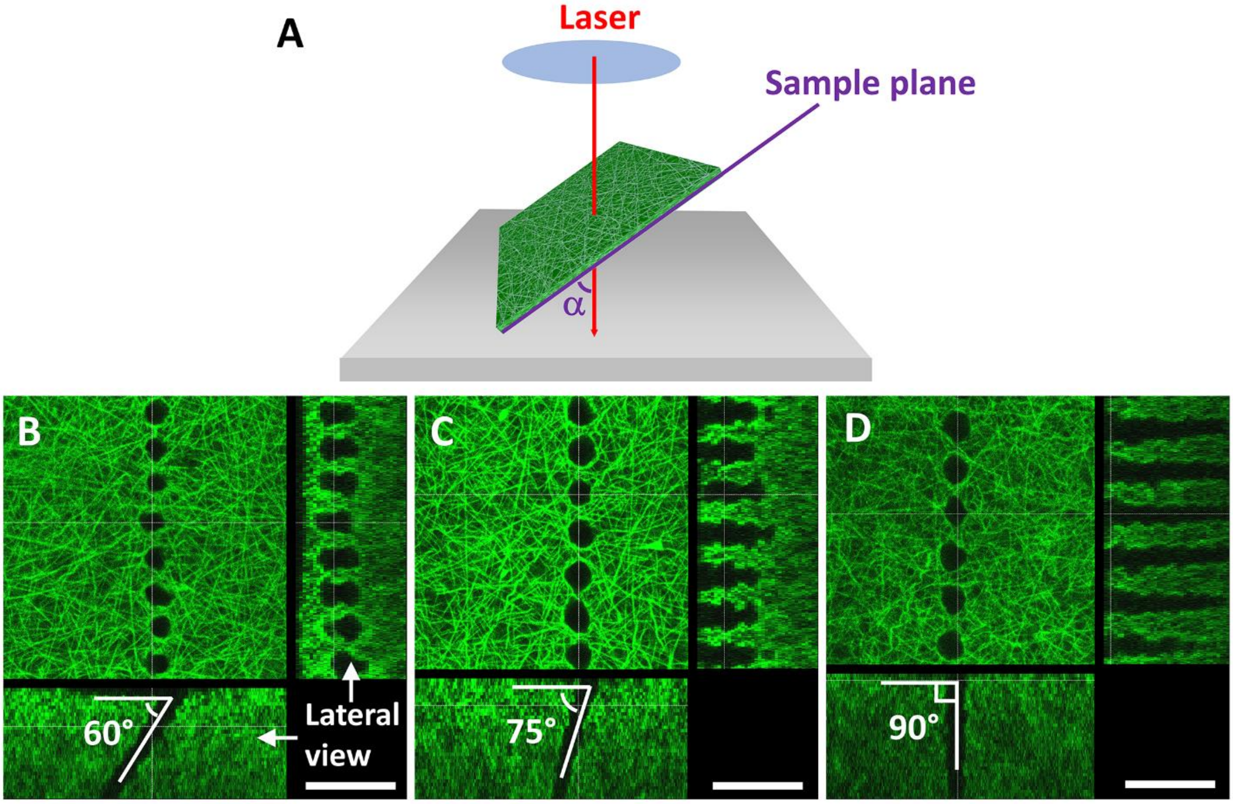

Figure S2. **(A)** Illustration of controlling the orientation of the tubules by adjusting the angles between the laser direction and the sample plane. (**B-D**) Confocal images of micropatterned tubules with different orientation angles to the matrix plane. **(B)** 60°, **(C)** 75°, and (**D)** 90°. Scale bars: 10 μm.

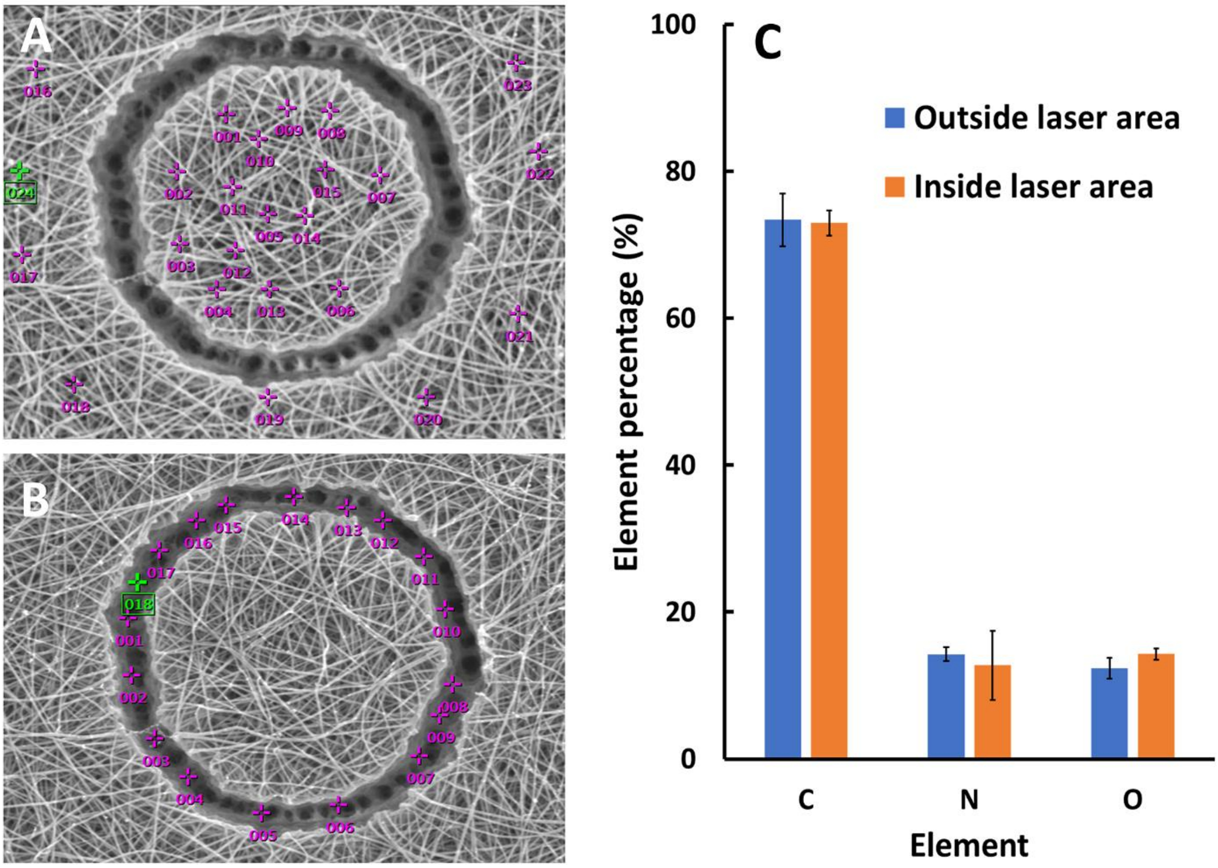

Figure S3. The element composition of the matrix surface outside and inside the laser-ablated area. The SEM images showed the spot measurements outside the laser areas (A) and inside the laser areas (B) to check whether the gelatin nanofibrous matrix endured compositional changes as a result of laser ablation, where the surface chemical composition was analyzed by Energy-Dispersive X-ray spectroscopy (EDX). Quantitative analysis was taken by counting the element compositions (carbon, nitrogen and oxygen) outside and inside the laser areas (C). No significant difference was observed for these elements on the nanofibrous matrix with and without the laser treatment ($p=0.16$, $n=50$).

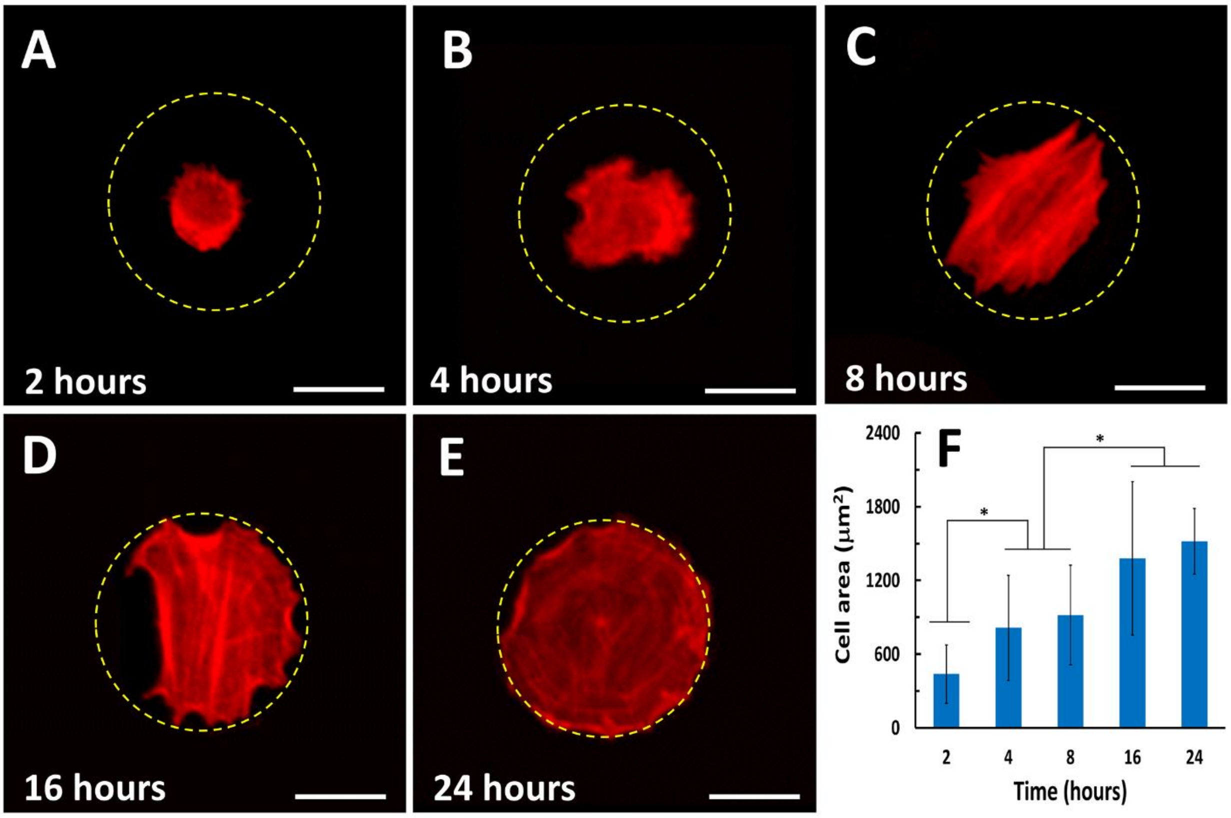

Figure S4. DPSC adhesion on the nanofibrous microislands. The morphology of the DPSC after seeded on the microisland for **(A)** 2 hours, **(B)** 4 hours, **(C)** 8 hours, **(D)** 16 hours, and **(E)** 24 hours. The F-actin of the DPSC was stained with red, and the yellow cycle dot shows the microisland. **(F)** The spreading area of the DPSCs at each time point (n= 20, *p< 0.05). Scale bars: 20 μm.

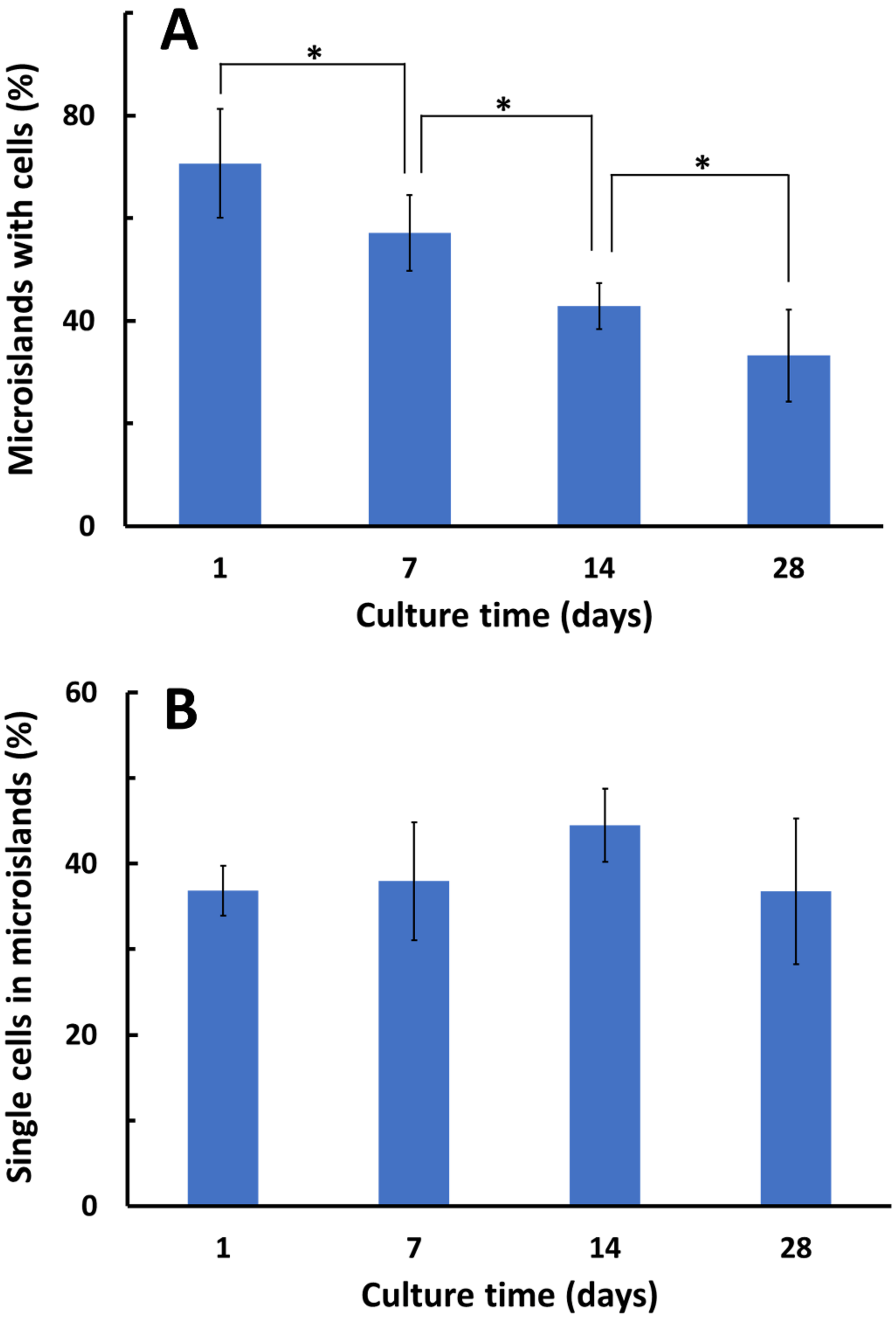

Figure S5. Cell viability after culturing the DPSCs on the microislands from 1 day to 28 days. **(A)** The changes of the ratios of the microislands occupied with living DPSCs when cultured from 1 day to 28 days. **(B)** The single cell ratios in the microislands when cultured from 1 day to 28 days. Five region of interests (ROIs) and at least 100 microislands in each ROI were selected in each time point for the analyses. *$p < 0.05$.

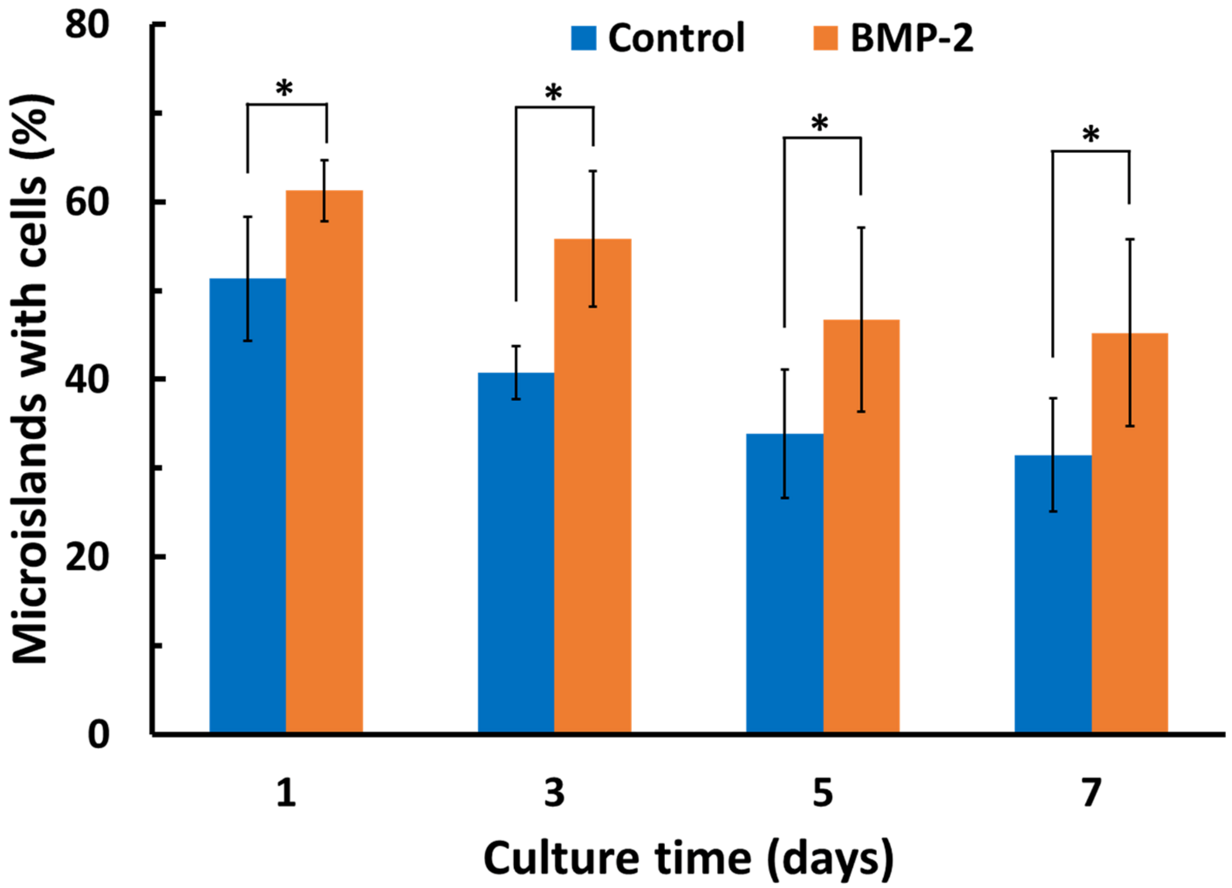

Figure S6. The comparison of DPSC viability cultured on microislands and BMP-2 conjugated microislands for 7 days. Five region of interests (ROIs) and at least 100 microislands in each ROI were selected in each time point for the analyses. *$p < 0.05$.

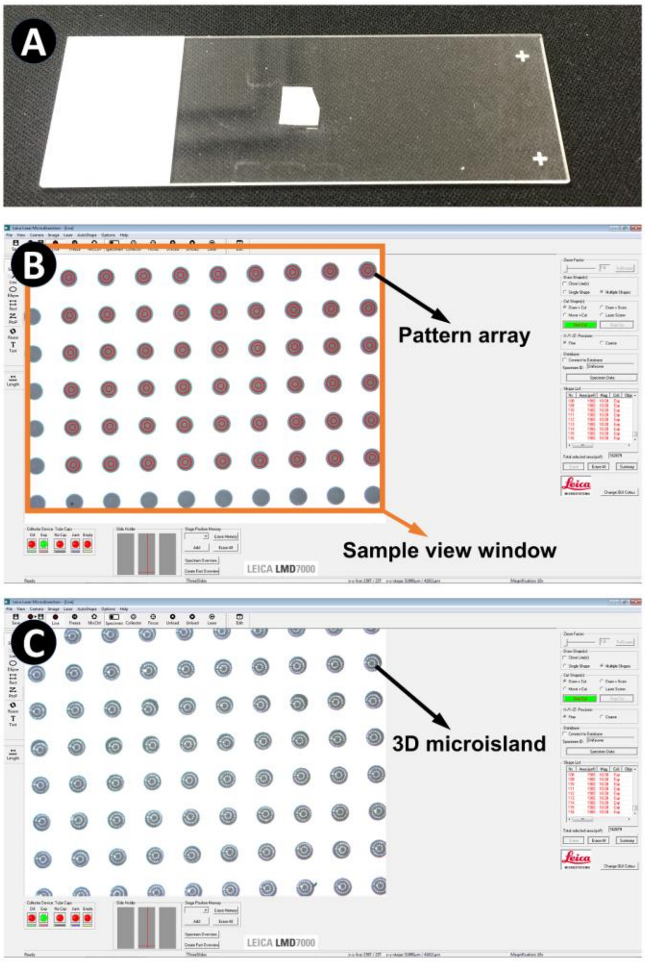

Figure S7. Fabrication of the tubular microislands. **(A)** The micropatterned matrix was dried and paved on a glass slide before the laser process. **(B)** The software window to show the creation of tubules within the microislands. Due to transparency of the nanofibrous gelatin matrix, the pattern of the microislands was readily visible under the optical microscope. The designed shape array (red cycle) was arrayed based on the cell island. **(C)** Optical images of the tubular microislands generated via laser ablation.

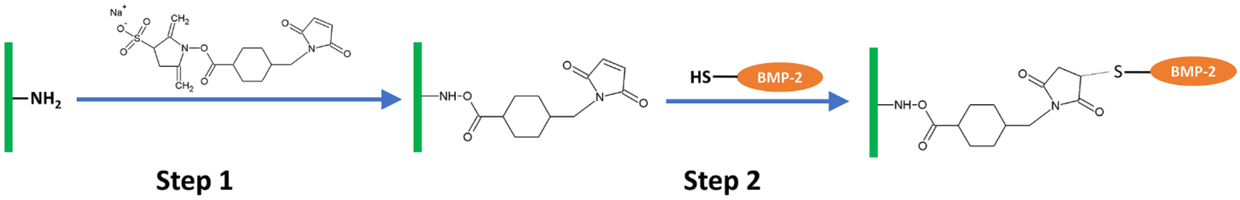

Scheme S1. Conjugation of BMP-2 to the nanofibers of microislands. In step 1, the NHS-ester groups reacted with the amino group (-NH2) on the GelMA nanofibers. In step 2, the maleimide groups reacted with the –SH group on the BMP-2.
